# A Quantitative Analysis of Social Media to Determine Trends in Brain Tumor Care and Treatment

**DOI:** 10.7759/cureus.11530

**Published:** 2020-11-17

**Authors:** Cylaina E Bird, Elliott D Kozin, Scott Connors, Christian LoBue, Kalil Abdullah

**Affiliations:** 1 Neurological Surgery, University of Texas Southwestern Medical Center, Dallas, USA; 2 Otolaryngology, Harvard Medical School, Boston, USA

**Keywords:** brain tumor, social media, glioblastoma, glioma, internet

## Abstract

Background

Approximately 80,000 primary brain tumors are diagnosed annually. Social media provides a source of information and support for patients diagnosed with brain tumors; however, use of this forum for dissemination of information about brain tumors has not been evaluated. The objective of this study was to evaluate social media utilization and content related to brain tumors with an emphasis on patients’ trends in usage.

Methods

Social media platforms were systematically evaluated using two search methods: systematic manual inquiry and a keyword-based social media tracker. The search terms included brain tumor, glioblastoma, glioma, and glioblastoma multiforme. Social media content (which includes Facebook pages and groups, YouTube videos, and Twitter or Instagram accounts) and posts were assessed for activity (as quantified by views of posts) and analyzed using a categorization framework.

Results

The manual and keyword searches identified 946 sources of social media content, with a total count of 7,184,846 points of engagement. Social media platforms had significant variations in content type. YouTube was the largest social media platform for sharing content related to brain tumors overall, with an emphasis on surgical videos and documented patient experiences. Facebook accounted for the majority of patient-to-patient support, and Twitter was the most common platform for scientific dissemination. Overall social media content was mostly focused on treatment overviews and patient experience. When evaluated by search term, most social media posts by the “brain tumor” community shared illness narratives, and searches specific to “glioma” and “glioblastoma” demonstrated a higher proportion of educational and treatment posts.

Conclusions

This study presents novel observations of the characteristics of social media utilization for the online brain tumor community. A robust patient community exists online, with an emphasis on sharing personal narratives, treatment information, patient-to-patient support, treatment options, and fundraising events. This study provides a window to the role of social media utilization by patients, their families, and health professionals. These findings demonstrate the different roles of Facebook, YouTube, and Twitter in the rapidly changing era of social media and its relationship with neurosurgery and neuro-oncology.

## Introduction

Patients diagnosed with primary brain tumors face challenges related to prognosis, treatment, and long-term outcomes. Patients with challenging illnesses use social support as a way to manage and address concerns about their illness [1]. With nearly 75% of Americans using YouTube, and a similar number active on Facebook, social media sites can provide individuals with a virtual community [2]. Patients have increasingly adopted the use of social media for educational purposes as well as emotional support for their healthcare concerns [2-6].

For healthcare professionals, social media can be used to bolster exposure, promote novel treatments and technologies, and share work. These functions facilitate communication and advance knowledge about patient health conditions. Recent studies have attempted to understand social media and to assess its impact on research [4]. In addition, the altimetric movement emphasizes the use of data from social media platforms as an assessment of the dissemination of research [7]. Social media is a powerful tool not only for patient education and support but also for enhancing visibility and utilization of research, especially for specific health topics, such as brain tumors. Thus, while investigation into social media impact on specific illnesses is increasing, there has not been an investigation into the utilization of social media regarding brain tumors.

In this study, we aim to evaluate social media utilization related to brain tumors, with a specific emphasis on glioblastoma. We also describe the current utilization of social media to inform the neurosurgery community of information available through these platforms and the types of messages patients with brain tumors convey via this forum. These results can assist in the discussion of resources for patient information, bolster patient-physician relationship, and obtain a more complete understanding of the relationship between social media, neurosurgery, and neuro-oncology.

## Materials and methods

General search strategy and data collection

Social media sites were systematically evaluated for online discussion about “brain tumors”, “glioma”, and “glioblastoma”. We assessed social media content (including pages, groups, videos, and accounts) and posts on social media that addressed brain tumors. Two methods were utilized during this study. The first method was a systematic manual search for social media content on popular social media platforms. In the second search, metrics generated by a social media tracking tool assessed active posts about brain tumors and glioblastoma. The social media sites assessed were Facebook, Twitter, YouTube, and Instagram.

Manual search

Keywords “brain tumor”, “glioblastoma” , “glioblastoma multiforme”, “glioma”, and “glioma brain tumor” with permutations of these words as modifiers were submitted via site-specific search. The returned social media content was filtered for relevance and for English language. The inclusion criteria for social media content were determined by activity parameters similar to those proposed by Saxena et al. [3]. These were utilized to ensure that the included content had sufficient engagement for analysis. Information on the number of members (individuals who subscribe to a page or group), likes (individuals who have positively viewed the page), views (number of times the video has been watched), and followers (people who subscribe to accounts or tweets) were collected for all content found. The inclusion criteria included the following:

1. Facebook group must have at least 10 members.

2. Facebook page must have at least 10 likes.

3. Instagram group must have at least 10 likes.

4. Twitter account must have at least 10 followers.

Social media tracking search

**Figure 1:**
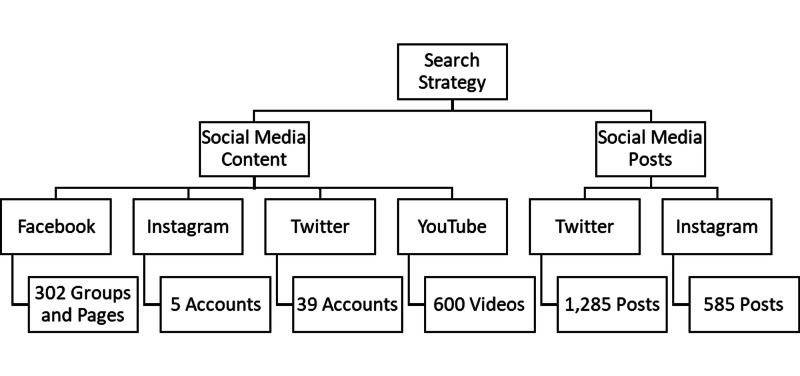
Social media content and posts meeting inclusion criteria

A social media tracking site (Keyhole Analytics LC) used the hashtags (#; demarcates a category or keyword) “brain tumor” and “glioblastoma” to identify active posts on social media sites, namely Twitter and Instagram (platforms in which the use of the “#” is prevalent). The social media tracker identified top posts, top linked domains, influential contributors, and commonly used keywords throughout the posts returned. Keyhole Analytics LC is a social media analysis platform that utilizes hashtags and keywords to evaluate social media sites with real-time, cross-sectional queries of the online community. This information is presented in both graphical and quantitative formats. One common form of graphical representation of data is a word cloud. Word clouds emphasize popular phrases or topics within a data sample and represent the frequency of utilization of a keyword or hashtag in the data set. The words that have a higher frequency of utilization are given a larger type size and a more central location in the cloud. Posts identified through the social media tracker were excluded from analysis if they were duplicates (such as retweets on Twitter or reposts on Instagram) or did not have content relevant to the diagnosis, treatment, management, or lived experience of an individual with a brain tumor. Figure 1 shows the social media content and posts that met the inclusion criteria.

Data categorization

A qualitative assessment of both the social media tracking and manual searches was conducted in order to identify major thematic discussions among patients. In addition, the most commonly cited educational sites or information were compiled. A sub-analysis was performed to discern the difference between the results in use and engagement for search term “brain tumor” compared to “glioblastoma”.

All the sources were verified by a member of the study team (C. E. B. and K. G. A.). The sources that did not meet the inclusion criteria were excluded from the data analysis. The included posts were categorized into one of eight groups. These groups are delineated in Table 1.

**Table 1:** Classification categories for social media content and posts

Classification Labels	Descriptions
Personal story	Posts detailing either a first or second-hand story about an individual’s journey through brain cancer or glioblastoma
General information/support	Posts that explained disease or the disease process, or provided general support for individuals with brain cancer or glioblastoma
Scientific research	Posts about scientific published research from a research group or scientific institution
Company/brand	Posts promoting or sponsored by a company/brand
News story	Posts from or about popular news sources regarding brain tumors
Treatment	Posts about treatment options or methods
Fundraiser/funding	Posts about fundraising events or searching for funding for treatment or research
other topic	Posts about a specific topic or question that do not fit in another category

Statistical analysis

Descriptive statistics were analyzed for all social media metrics. All analysis was performed using Excel 2016 (Microsoft Corporation, Redmond, WA, USA). Data were also qualitatively analyzed as described above.

Ethical considerations

This study was considered exempt from review by the University of Texas Southwestern Medical Center Review Board. Data collected for this study were accessed using publicly available social media sites including Facebook, Twitter, YouTube, and Google. There were no interactions with these social media sites or with the individual users themselves.

## Results

Manual search

**Figure 2:**
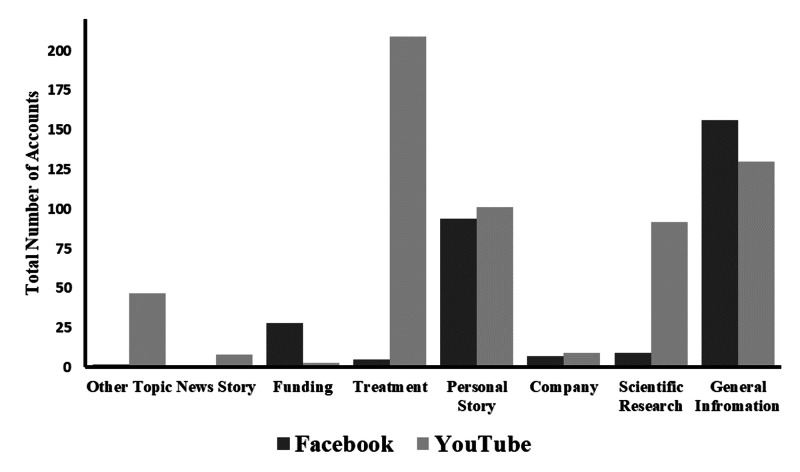
Facebook and YouTube content categorization by number of accounts or videos

The manual search identified a total of 946 sources of social media content accounts across the four social media sites analyzed. Descriptive statistics for these sources are summarized in Table 2. Figure 2 displays the distribution of content on the two most populous sites analyzed in the manual search, Facebook and YouTube.

**Table 2:** Descriptive statistics for manual search IQR, interquartile range

Social Media Platform	Total Likes/Followers/ Views	Average Number of Likes/Followers/Views	Median (IQR)
YouTube	6,343,677 views	10,573 views	1,100 views (387-5175)
Facebook	796,643 likes	2,638 likes	434 likes (134-2106)
Twitter	42,642 followers	1,093 followers	822 followers (209-1948)
Instagram	1,884 likes	377 likes	357 likes (30-734)

YouTube 

The 600 YouTube videos identified had a total of 6,343,677 views (median: 1,100; interquartile range [IQR]: 387-5175) and accounted for 63% of the social media content assessed. Most of the videos discussed treatment options. Personal stories, which were shared using personal video blogs, were the second most popular video category. The three most commonly viewed videos were entitled “DIPG Tumor (Kayne Episode 3)”, “Surgical Footage of Removal of Glioblastoma Multiforme (GBM) Brain Tumor (brain surgery)”, and “Signs and Symptoms of a Brain Tumor l Dana-Farber Cancer Institute”.

Facebook

The 151 Facebook groups and 151 Facebook pages identified amassed a total of 796,643 “Likes” (median: 434; IQR: 134-2106). The top Facebook groups were about personal stories including, “Prayers for Sophie”, “Get well Gabby Foundation”, and “Richards Journey”.

Twitter and Instagram

There were 39 Twitter accounts and 5 Instagram accounts identified in the manual search. Of the Twitter accounts identified, 40% of the accounts were dedicated to presenting scientific research. The majority of Instagram accounts were dedicated to general information/support related to glioblastoma (40%).

Social media tracking search

**Figure 3:**
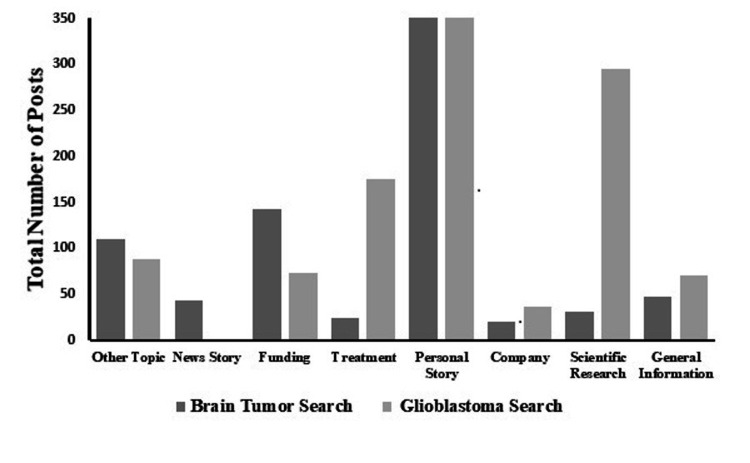
Altmetrics search post categorization for brain tumor and glioblastoma searches by total number of posts

While the accounts identified through the manual search aided in the description of accessible social media content on common social media sites, a social media tracker was able to identify specific posts that were related to the keywords “brain tumor” or “glioblastoma”. Figure 3 displays the distribution of categories identified by the social media posts.

“Brain tumor” tracking search

**Figure 4:**
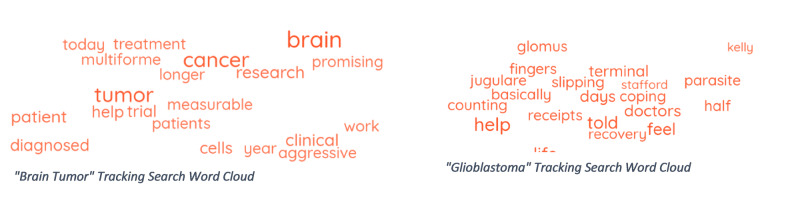
Keyword tracking search word clouds

“Brain tumor” was mentioned as a keyword in 18,376 posts. However, manual verification led to the inclusion of 688 of the 18,376 (3.7%) posts. Of the included social media posts, 52% were used to describe personal stories. The most linked domain recognized was ESPN™ related sites, with the second most common site being GoFundMe™ pages. The keywords with the highest amount of online activity are displayed in the word cloud in Figure 4. Educational institutions including the University of Southern California, Cleveland Clinic, Medical College of Wisconsin, and Mayo Clinic posted relevant material during the time of tracking. However, social media posts from educational institutions were overall poorly represented in this search.

“Glioblastoma” tracking search

Of the 1,678 posts selected, 1,180 (70%) were included in the analysis. Many of the posts identified discussed information about fundraisers and treatment (36%). The most linked domain in this search was Nature.com followed by other sites that discussed research articles about advances in glioblastoma diagnosis and treatment. Educational institutions identified in this search included Cleveland Clinic, University of California, Los Angeles, University of Texas at Dallas, Brigham and Women’s Hospital (Harvard Medical School), and University of Southern California.

## Discussion

Social media is an increasingly common way to learn, discuss, and share information [1,3-4,8]. Previous studies have analyzed social media utilization in brain aneurysms, vagal nerve stimulators placement, and pediatric hydrocephalus, but, to our knowledge, there has not been an evaluation of social media utilization for brain tumors, particularly glioblastoma [1,9-14]. Our data demonstrate that there is a robust community of individuals using the internet as a communication tool. These individuals and institutions use social media sites to share content and posts that support, inform, and educate regarding brain tumors and their care.

In this study, each social media platform occupies a distinct niche within the online environment, which was evident in both qualitative and quantitative assessment. YouTube has the largest collection of social media content dedicated to discussing brain tumors, as it had the most recorded activity, particularly regarding glioblastoma. The videos focus on both technical aspects of care delivery as well as testimonies documenting patient experiences. Although it is unclear whether the procedural videos are intended mainly to demystify the surgical process for patients or to share technical nuance between surgeons, in the analysis, YouTube was a highly utilized content source. Comparatively, Facebook’s content skewed toward patient-to-patient support groups and seemed to function as a surrogate for in-person community support. Given the relative rarity of glioblastoma and Facebook’s role as a communication platform, this is a logical extension from local support and information dissemination.

Twitter, with its short character limit and real-time release capabilities, was the most common site per post for content related to research articles and initiatives [7]. Most frequently, there were links or redirections to currently published scientific articles, scientific advances, or new patient resources. Twitter, along with Instagram, had the most commonly updated and the most frequent “daily” postings of novel information. This again is consistent with the goal of these two platforms, which is facile dissemination of novel information.

While scientific information to and from surgeons and scientists was a common finding, especially on Twitter, our data show that most of the social media posts documented personal stories from individuals with a brain tumor, while other forms of social media content emphasized general information and support. Previous work has demonstrated that personal story posts fall into one of six domains [15]. These domains include “inspiration and motivation, providing and sharing information, requesting information, seeking emotional support and well wishes, admiration, and loss and grief” [15]. The personal story posts identified in this study followed a similar pattern. Included in this are stories from celebrities who address having brain tumors. For instance, one prominent topic in posts was the wife of an NFL football player who publicly discusses her surgery for an acoustic neuroma. This was likely the reason for ESPN being a prominently linked domain in posts in this study. This highlights a similarity between the online brain tumor community and other media domains where high-profile individuals can drastically influence exposure [16].

Comparatively few scientific research articles and posts from educational or healthcare institutions were analyzed in this study. Our analysis demonstrated that using the more specific search category of “glioblastoma” compared to “brain tumor” yields a more substantial number of scientific research posts and posts from educational institutions. These posts shared many details about treatment options and had a firmer basis in peer-reviewed publications than those proposed by other users. This data emphasize the value of circulating educational information on social media as many patients use the internet to research their diagnoses. It also reflects an opportunity to improve outreach from healthcare providers and institutions to patients in search of reliable information.

Finally, illustrating the difficulty patients may face in affording comprehensive neuro-oncologic care, fundraising posts were found mostly on Twitter with links to personal GoFundMe pages or to support events where funds were donated for future research.

Limitations

Similar to other studies on social media utilization, this study used social media platforms and a keyword tracker to identify relevant posts. This limits the results that could be evaluated as content creators may use other specifiers to create their online support and education systems. Those not using the specified terms were not assessed in this study. Another limitation of using a social media tracker during a specific time frame is that it provides a cross-sectional analysis. Therefore, there may be more comments posted before or after the data collection period that represent other categories important in assessing social media utilization for the brain tumor community. All the information gathered in this study was publicly available; therefore, private sites were not assessed. Social media platforms are constantly evolving; thus, there may be a difference in utilization based upon the user. However, these data were not assessed during this study.

## Conclusions

In this study, we describe and analyze the use of social media for the online brain tumor community. Our results describe a robust online community with posts and accounts dedicated to the topic of brain tumors, especially glioblastoma. This community is spread over several social media platforms, with each social media platform occupying a particular role in this environment based on the structure of information sharing. The natural division of these roles is a digital example of form-fitting function, with different social media sites being used to create social support groups, provide education on brain tumors, document the illness experience from the patient perspective, and inform regarding treatment options. This perspective assists in appreciating the relationship between social media, neurosurgery, and neuro-oncology. Understanding the material currently available in these online communities and the opportunities for their use will help healthcare providers better guide their patients who use social media as a supportive and educational healthcare resource and may aid patient-physician communication and relationships.
