# Correction: A Quantitative Analysis of Social Media to Determine Trends in Brain Tumor Care and Treatment

**DOI:** 10.7759/cureus.c44

**Published:** 2021-06-14

**Authors:** Cylaina E Bird, Elliott D Kozin, Scott Connors, Christian LoBue, Kalil Abdullah

**Affiliations:** 1 Neurological Surgery, University of Texas Southwestern Medical Center, Dallas, USA; 2 Otolaryngology, Harvard Medical School, Boston, USA

This article was originally published with Figure 2 appearing twice (as both Figure 2 and Figure 3). The correct Figure 3 has now been uploaded to the article. Cureus regrets this error.

